# Angiopathic activity of LRG1 is induced by the IL-6/STAT3 pathway

**DOI:** 10.1038/s41598-022-08516-2

**Published:** 2022-03-22

**Authors:** Athina Dritsoula, Laura Dowsett, Camilla Pilotti, Marie N. O’Connor, Stephen E. Moss, John Greenwood

**Affiliations:** Institute of Ophthalmology, University College London, 11-43 Bath Street, London, EC1V 9EL UK

**Keywords:** Cell biology, Molecular biology

## Abstract

Leucine-rich α-2-glycoprotein 1 (LRG1) is a secreted glycoprotein that under physiological conditions is produced predominantly by the liver. In disease, its local induction promotes pathogenic neovascularisation while its inhibition leads to reduced dysfunctional angiogenesis. Here we examine the role of interleukin-6 (IL-6) in defective angiogenesis mediated by LRG1. IL-6 treatment induced LRG1 expression in endothelial cells and ex vivo angiogenesis cultures and promoted vascular growth with reduced mural cell coverage. In *Lrg1*^−/−^ explants, however, IL-6 failed to stimulate angiogenesis and vessels exhibited improved mural cell coverage. IL-6 activated *LRG1* transcription through the phosphorylation and binding of STAT3 to a conserved consensus site in the *LRG1* promoter, the deletion of which abolished activation. Blocking IL-6 signalling in human lung endothelial cells, using the anti-IL6 receptor antibody Tocilizumab, significantly reduced *LRG1* expression. Our data demonstrate that IL-6, through STAT3 phosphorylation, activates *LRG1* transcription resulting in vascular destabilisation. This observation is especially timely in light of the potential role of IL-6 in COVID-19 patients with severe pulmonary microvascular complications, where targeting IL-6 has been beneficial. However, our data suggest that a therapy directed towards blocking the downstream angiopathic effector molecule LRG1 may be of greater utility.

## Introduction

Leucine-rich α-2-glycoprotein 1 (LRG1) is a member of the conserved leucine-rich repeat family of secreted glycoproteins involved in a plethora of physiological processes^1,2^. Under normal conditions, LRG1 is synthesised almost exclusively by the liver from where it is secreted into the circulation as a purported acute phase-like protein^3,4^. However, in many conditions, including viral infections, diabetes, autoimmune and cardiovascular disease, fibrosis, and cancer, LRG1 levels are elevated both locally and systemically^5–13^. In such cases, LRG1 has been characterised as a pathogenic factor implicated in disease progression and proposed as both a potentially useful diagnostic and prognostic biomarker, and therapeutic target^14^. Growing evidence emerging from these studies also points to LRG1 being a novel proangiogenic factor that through local upregulation contributes to the formation of defective vessels. This is currently most evident in the eye and kidney, where it plays a central role in the vascular dysfunction observed in models of retinal neovascularisation and diabetic kidney disease and is consistent with raised LRG1 levels in patients with these diseases^15–17^. Moreover, in cancer, where there is an emerging body of evidence for a pathogenic role for LRG1^14,18^, the induction of LRG1 in tumour endothelial cells has also been shown to contribute to pathological neovascularisation^19^ and priming the vascular metastatic niche^20^ establishing this secreted glycoprotein as a new vasculopathic molecule.

Pathological angiogenesis is a complex process characterised by reduced mural cell coverage, vascular leakage, and dysfunctional perfusion. Mural cells, in particular pericytes, exhibit a broad phenotype depending on their position in the vascular hierarchy and cues from the surrounding microenvironment, and play a vital role in the induction and maintenance of microvascular integrity^21,22^. Endothelial and mural cell interactions are critical for vascular homeostasis, and disruption of this intimate relationship has been implicated in the pathogenesis of various diseases. A number of key signalling processes have been identified as being central to vessel maturation and stability, including perivascular cell-derived growth factors and their receptors such as angiopoietins/Tie1-2, VEGF/VEGFR, TGFβ/TGFβR1-2, PDGF/PDGFR, S1P/EDG1, as well as the Notch family ligands^23^. Associated with disturbances in these signalling pathways is disruption of the close spatial proximity between pericytes and endothelial cells and this is most evident in the tumour environment^24^. In this context, we have shown that LRG1 is a causal factor in disrupting the endothelial-mural cell association in various tumour models and that antibody blockade or genetic deletion of *Lrg1* results in improved mural cell coverage and vascular function^19^. Consistent with this, tumour vessels exhibited reduced vascular leakage, increased perfusion, and enhanced chemotherapeutic and immunotherapeutic efficacy^19^. This evidence collectively establishes LRG1 as an important vascular destabilising factor and illustrates its potential as a therapeutic target that may be of significant relevance to diseases where vascular dysfunction is problematic.

How LRG1 contributes to vascular destabilisation is beginning to emerge. Recent studies in the eye, kidney, and brain have established that LRG1 exerts its defective angiogenic effects in part through changing the balance in endothelial cells of the TGFβ pathway in favour of the ALK1-SMAD1/5/8 proangiogenic signalling arm^14–16^. Thus, LRG1 may be an important factor in driving the angiogenic switch in TGFβ signalling. The upstream mechanisms through which LRG1 is induced under disease conditions, however, remain poorly defined with studies so far focusing predominantly on its post-transcriptional and post-translational regulation^25–28^. Nonetheless, evidence has emerged that proinflammatory cytokines, such as IL-6 and TNFα, are capable of *LRG1* induction^4,29–32^. Such cytokines have already been established as mediators of vascular dysfunction that contribute to neovascularisation^33^, and IL-6 has recently been shown to induce defective angiogenesis in a VEGF-independent manner^34^. Furthermore, IL-6 has been proposed as a contributing factor to diabetic eye disease^35,36^ and in ovarian cancer as a possible mechanism of resistance to anti-VEGF treatment^37^. These proangiogenic IL-6 properties have recently been brought into sharp focus in light of the central role inflammatory cytokines play in the devastating vasculopathy associated with severe acute respiratory syndrome coronavirus 2 (SARS-CoV-2) infection. How proinflammatory cytokines disturb the vasculature is multifactorial, but these relationships suggest that some of the disruptive vascular effects may be mediated through cytokine induction of LRG1. Consistent with this possibility, circulating LRG1 levels have been reported to be raised in severe COVID-19 patients^38–41^ and in patients with vasculitis^42,43^, where vascular damage is a primary feature.

To address these possibilities, we investigated the involvement of IL-6 in the transcriptional regulation of *LRG1* and determined whether LRG1 mediates the reported IL-6-driven vascular destabilising effects. We show that the IL-6-STAT3 pathway is an activator of *LRG1* transcription and that inhibition of IL-6 results in reduced *LRG1* production. We propose, therefore, that direct targeting of LRG1 may be an alternative, or complementary, therapy for COVID-19-related pulmonary microvascular complications and other disease where the vasculature is disrupted by IL-6.

## Results

### LRG1 induces angiogenesis with reduced mural cell coverage

**Figure 1:**
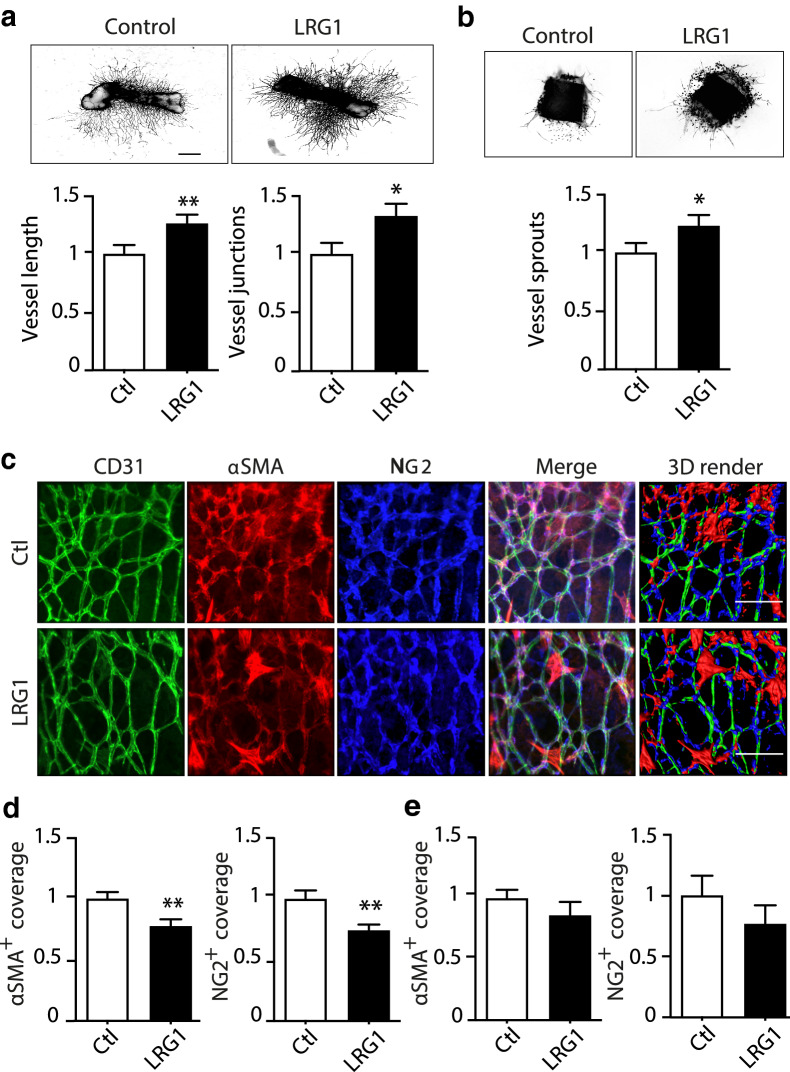
LRG1 treatment induces angiogenesis with reduced mural cell coverage. Mouse metatarsal bones (**a**) and aortic rings (**b**) were treated with PBS as control (Ctl) or 70 μg/ml recombinant human LRG1 and analysed for angiogenesis and sprouting respectively. (**c**,**d**) Representative images of metatarsal bones treated with PBS as control (Ctl) or 70 μg/ml LRG1 and stained with antibodies against CD31 (green), NG2 (blue), and αSMA (red) for mural cell coverage and analysis. (**e**) Mural cell coverage analysis for aortic rings. Scale bars, 50μm. N ≥ 3 independent experiments. Student’s t-test, Mean ± SEM, * ≤ 0.05, ** ≤ 0.01.

To evaluate the potential contribution of LRG1 to neoangiogenesis, we treated ex vivo cultures of metatarsal bones and aortic rings with recombinant human LRG1. LRG1 induced a significant increase in total vessel length and junctions in the metatarsal bone assay (Fig. 1a), as well as sprouting in the aortic ring assay (Fig. 1b), as previously reported^15^. Since LRG1 exhibits angiopathic properties and may interfere with the delicate interplay between the endothelium and mural cells^19^, we next investigated whether LRG1 altered this association. Angiogenic explant cultures were treated with exogenous LRG1, which resulted in a significant reduction in αSMA^+^ and NG2^+^ mural cell coverage of vessels in the metatarsal bone assay (Fig. 1c,d). A reduction was also observed in the aortic ring cultures, but this did not reach significance (Fig. 1e). These data support a role for LRG1 in promoting pathological angiogenesis, possibly through interfering with the close endothelial-mural cell association.

### IL-6 upregulates LRG1 expression

**Figure 2:**
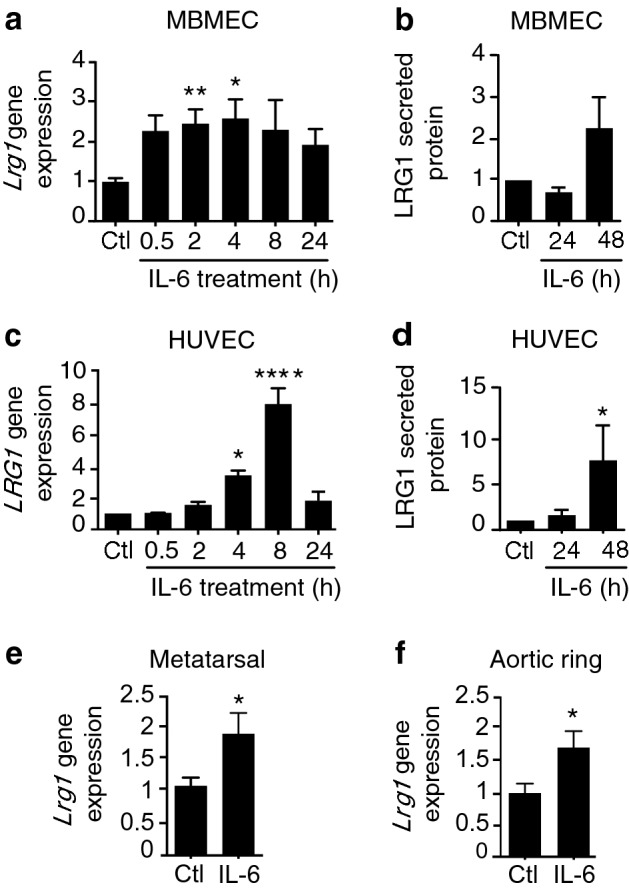
IL-6 upregulates LRG1 gene and protein expression. Mouse brain microvascular endothelial cells (MBMEC) (**a**,**b**) and HUVEC (**c**,**d**) were treated with PBS as control (Ctl) or 50 ng/ml IL-6 for a time course over 24 and 48 h. *LRG1* gene expression was assessed by RT-qPCR (**a**,**c**), and secreted LRG1 protein was assessed by western blotting following acetone precipitation and normalisation to amount of total protein by Ponceau S staining (**b**,**d**). ANOVA with multiple comparisons test (Dunnett’s). Mouse metatarsal bones (**e**) and aortic rings (**f**) obtained from wild type animals were cultured and treated with PBS as control (Ctl) or 50 ng/ml IL-6. RNA was extracted and used for RT-qPCR. Human and mouse *LRG1* gene expression were normalised to housekeeping genes. Mann Whitney test. N ≥ 3 independent experiments. Mean ± SEM, * ≤ 0.05, ** ≤ 0.01, **** ≤ 0.0001.

Having established that LRG1 corrupts the angiogenic process, we next investigated whether IL-6 is implicated in LRG1-driven pathogenic angiogenesis by assessing its effect on *LRG1* gene expression. Our previous work has shown that in neovascular complications of the retina, *Lrg1* expression is restricted to the neovascular endothelial cells^15,44^, and in LRG1-negative tumours its expression is restricted to the tumour neovascular endothelial cells^19^. Consistent with these previous data, low levels of *Lrg1* gene expression were observed in cultured mouse brain microvascular endothelial cells (MBMEC), while the *Lrg1* transcript was not detectable in pericytes from the same source (Supplementary Fig. 1a). Next, we tested whether IL-6 can induce LRG1 expression in various types of endothelial cells (MBMEC, human umbilical vein endothelial cells (HUVEC) and human pulmonary microvascular endothelial cells (HPMEC)). IL-6 significantly increased the gene expression of *LRG1* in the endothelial cells as well as the secreted LRG1 protein, albeit with slightly different kinetics (Fig. 2a–d, Supplementary Fig. 1b, Supplementary Fig. 2). Similar effects were also seen in the ex vivo cultures of mouse metatarsal bones and aortic rings treated with IL-6 for a total of 11 or 7 days, respectively (Fig. 2e,f). IL-6, however, did not induce *Lrg1* expression in mouse brain pericytes (Supplementary Fig. 1c).

### IL-6 induces LRG1-dependent defective angiogenesis

**Figure 3:**
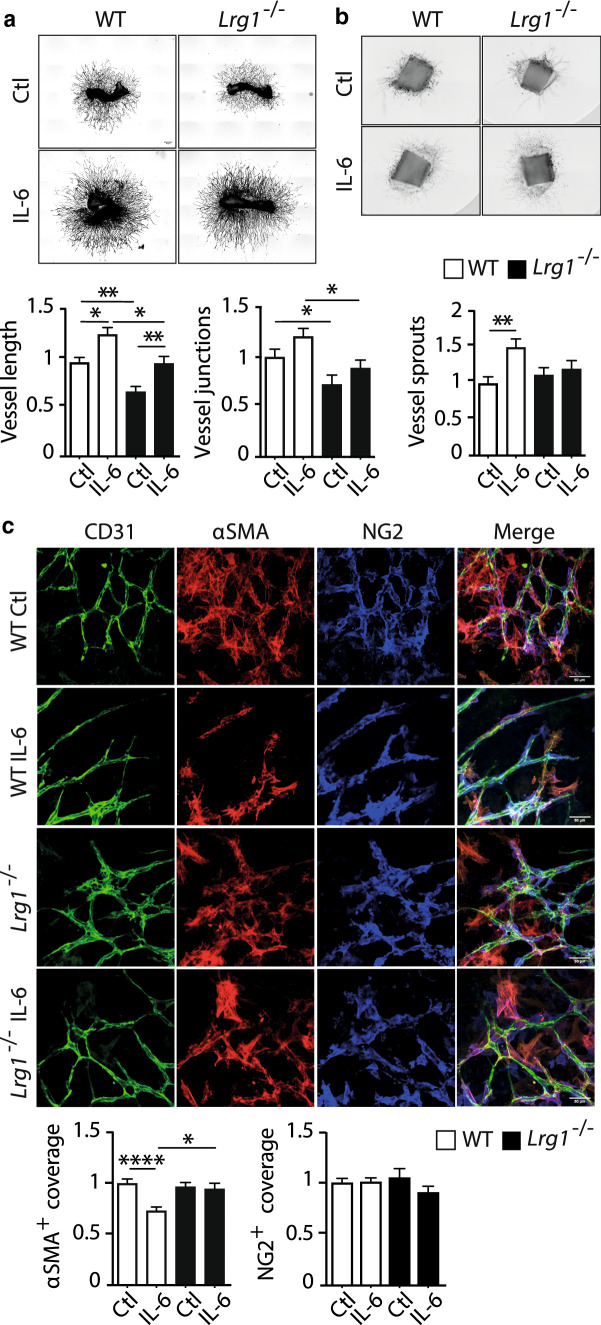
IL-6 induces LRG1-dependent defective angiogenesis. Mouse metatarsal bones (**a**) and aortic rings (**b**) from wild type C57Bl6 and *Lrg1*^−/−^ mice were cultured and treated with 50 ng/ml IL-6 and analysed for angiogenesis and sprouting respectively. (**c**) Representative images of metatarsal bones treated with PBS as control (Ctl) or 50 ng/ml IL-6 and stained with antibodies against CD31 (green), NG2 (blue), and αSMA (red) for mural cell coverage and analysis. Scale bars, 50μm. N ≥ 3 independent experiments. ANOVA with multiple comparisons test (**a**–**c**: Dunn’s, **b**: Tukey’s), Mean ± SEM, * ≤ 0.05, ** ≤ 0.01, **** ≤ 0.0001.

After showing that IL-6 is an upstream activator of LRG1 expression in endothelial cells and in angiogenic explants, and since both are involved in dysfunctional angiogenesis, we next examined whether previously reported defective angiogenesis driven by IL-6^34^ was mediated by downstream LRG1. Mouse metatarsal and aortic ring tissues were prepared from wild type and *Lrg1*^−/−^ animals and treated with IL-6 over 7–11 days. Genetic deletion of *Lrg1* reduced vascular growth in the metatarsal assay as measured by vessel length and junctions (Fig. 3a). Conversely, IL-6 treatment increased total vessel length compared to vehicle-treated cultures (Fig. 3a). However, in the *Lrg1*^−/−^ metatarsals, IL-6-independent and -dependent vascular growth were both significantly reduced (Fig. 3a), highlighting that LRG1 is indispensable for these IL-6-driven effects in this model. Similarly, in the aortic ring assay, IL-6 significantly increased vessel sprouting in wild type explants but failed to induce significant angiogenesis in the absence of *Lrg1* (Fig. 3b).

A previous study has reported reduced pericyte coverage in response to IL-6 when compared to VEGF^34^. We therefore investigated whether the effect of IL-6 on perivascular cell coverage was also mediated through LRG1. Data from the metatarsal assay showed that IL-6 treatment caused a significant reduction in αSMA^+^ mural cell coverage, which was restored in the absence of the *Lrg1* gene (Fig. 3c), suggesting that LRG1 is a downstream effector molecule of IL-6 mediated defective vessel formation. However, the NG2^+^ perivascular cell population was not affected by IL-6 treatment or the genetic ablation of *Lrg1*, revealing differences in mural cell subpopulations and suggesting that LRG1 is particularly important for αSMA^+^ expressing mural cells (Fig. 3c). In contrast to the metatarsal assay, IL-6 did not cause reduced αSMA^+^ mural cell coverage in wild type aortic ring explants, and consistent with this, IL-6 had no effect in explants from *Lrg1* null mice (Supplementary Fig. 3). It is important to note here that mural cells express, if any, very low levels of IL-6 receptor compared to the endothelial cells depending on the surrounding microvasculature (Supplementary Fig. 4). These data highlight differences in the regulation of angiogenesis and mural cell coverage by IL-6 and LRG1 that are dependent on the vascular bed, the pattern of mural cell expression, and the complexity of the multicellular environment in these explants.

It has been previously reported that IL-6-driven angiogenesis with reduced pericyte coverage is partly mediated through Notch and angiopoietin signalling^34^. To study whether these two pathways are also involved in LRG1-dependent angiogenesis as downstream effectors in addition to the TGFβ pathway, transcriptional analysis was performed on metatarsal cultures treated with IL-6. These data showed no difference in the levels of *Angpt1*, but a significant upregulation was seen in the *Angpt2* gene following IL-6 treatment, which was diminished when *Lrg1* was genetically ablated (Supplementary Fig. 5). In addition, expression of *Hey1* was significantly reduced in the wild-type IL-6-treated cultures and those obtained from *Lrg1*^−/−^ animals (Supplementary Fig. 5), but no difference was seen for *Hes1*, *Dll4*, and *Jagged1*.

### IL-6 activates *LRG1* gene transcription

**Figure 4:**
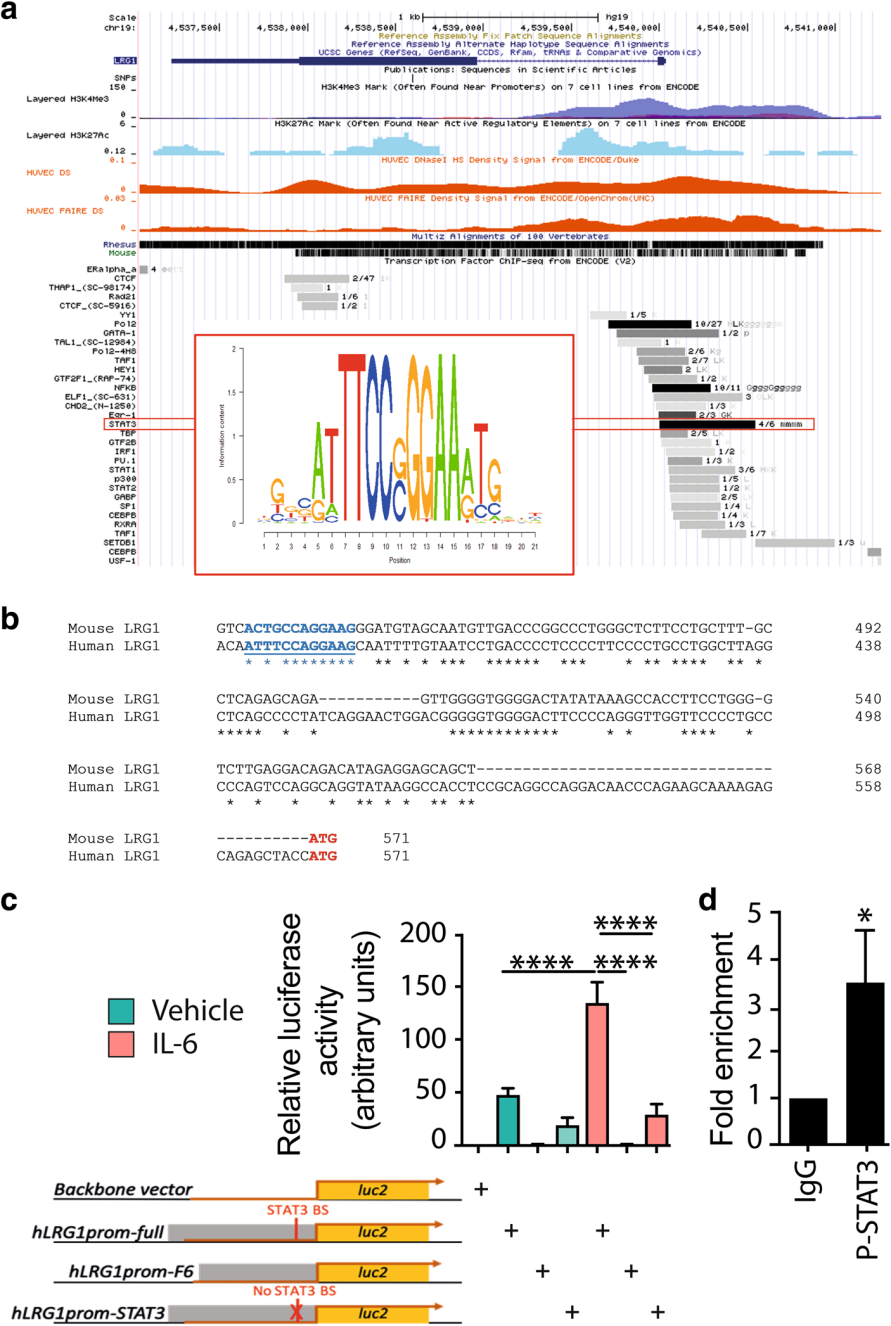
IL-6 activates *LRG1* gene transcription. (**a**) *In-silico* analysis of the human *LRG1* gene promoter showing transcription factor binding predictions, including the STAT3 consensus binding site (UCSC genome browser). (**b**) Nucleotide alignment (ClustalW) of the mouse and human *LRG1* gene promoters. The STAT3 consensus binding site is underlined and highlighted in blue, and the translation initiation site is highlighted in red. (**c**) Luciferase reporter assays performed in HepG2 cells stimulated with PBS as control/vehicle or 50 ng/ml IL-6 and transfected with luciferase plasmids. Backbone vector: pGL4.10 plasmid, hLRG1prom-full: pGL4.10 with the full-length human *LRG1* promoter driving expression of the luciferase (*luc2*) gene, hLRG1prom-F6: hLRG1prom-full without 200 bp surrounding the STAT3 binding site, hLRG1prom-STAT3, hLRG1prom-full without 54 bp surrounding the STAT3 binding site. ANOVA with multiple comparisons test (Tukey’s), (**d**) Chromatin immunoprecipitation (ChIP) assays performed in HepG2 cells stimulated with 50 ng/ml IL-6 for 16 h. An anti-P-STAT3 antibody (cs #9145) was used for the ChIP, and a normal rabbit IgG (cs #2729) was used as a negative control. Data are presented as fold enrichment to IgG (2^-ΔΔCt). Mann Whitney test. N ≥ 3 independent experiments. Mean ± SEM, * ≤ 0.05, **** ≤ 0.0001.

After establishing that IL-6-driven defective angiogenesis is mediated through LRG1, we sought to investigate this further at the transcriptional level. IL-6 signalling is complex as it involves two different pathways: the classic pathway, where IL-6 binds to the membrane-bound IL-6 receptor (IL-6R), and the trans-signalling pathway, where IL-6 binds the soluble receptor^45^. In both cases, binding of IL-6 to the receptor induces homodimerisation of the membrane-spanning protein IL-6 receptor subunit-β, also known as gp130, to further initiate signalling through the JAK/STAT pathway^45^. An *in-silico* analysis of the human *LRG1* gene promoter showed that among other transcription factors, STAT3, which is a central mediator of IL-6 signalling, is predicted to bind the human *LRG1* promoter in the open chromatin state (evidence based on DNase Hypersensitivity, FAIRE) to regulate and activate transcription (evidence based on H3K4Me3 and H3K27Ac signatures) (Fig. 4a). Nucleotide alignment of the mouse and human *LRG1* gene promoters showed that the consensus binding site of STAT3 is highly conserved (~ 83.3%) between the two species (Fig. 4b), further pointing to the potential importance of STAT3 binding on the gene promoter.

To study the transcriptional regulation of the *LRG1* gene driven by the IL-6/STAT3 signalling axis, luciferase reporter assays were performed. In HepG2 reporter cells, where gene and protein expression of LRG1 is driven by IL-6 (Supplementary Fig. 6), IL-6 stimulation significantly increased the transcriptional activity of the *LRG1* full-length promoter compared to unstimulated cells (Fig. 4c). However, this effect was abolished when the STAT3 binding site was deleted (Fig. 4c), providing evidence that IL-6 can activate *LRG1* transcription through the STAT3 transcription factor. Chromatin immunoprecipitation assays confirmed that phosphorylated STAT3 binds the human *LRG1* promoter upon IL-6 stimulation at the consensus binding site to activate gene transcription as shown by significant enrichment of binding (Fig. 4d). As a complementary approach, the JAK2-specific inhibitor AG490 was used to block the JAK/STAT signalling axis^46^ and confirm the involvement of STAT3 in *Lrg1* transcriptional regulation. IL-6 mediated phosphorylation of STAT3 was indeed blocked when MBMEC were treated with AG490, establishing the effectiveness of this pharmacological inhibition (Supplementary Fig. 7a). Consequently, the IL-6-driven *Lrg1* upregulation was also blocked by AG490 (Supplementary Fig. 7b). Collectively, these data show that upon IL-6 stimulation, phosphorylated STAT3 binds the *LRG1* gene promoter to initiate transcription.

### Therapeutic IL-6R blocking antibody Tocilizumab attenuates *LRG1* gene expression

**Figure 5:**
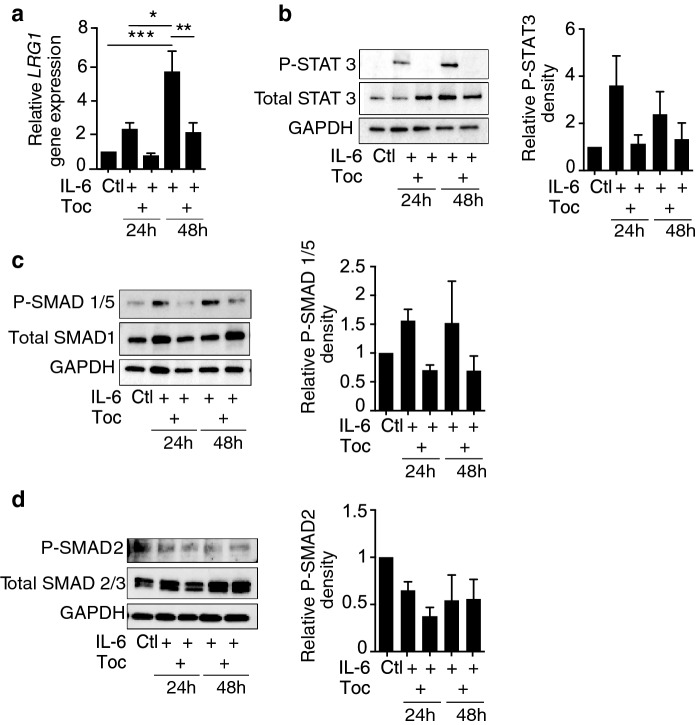
Tocilizumab antibody blocks *LRG1* gene expression. HPMEC cells treated with PBS as control (Ctl), 50 ng/ml IL-6, and 20 μg/ml Tocilizumab (Toc) antibody in combination with IL-6 as indicated. RNA and protein were extracted and subjected to RT-qPCR and western blotting. (**a**) Human *LRG1* gene expression normalised to *HPRT* housekeeping genes. Protein expression of phosphorylated and total STAT3 (**b**), SMAD1/5 (**c**) and SMAD2 (**d)**. Quantification was performed by densitometric analysis of normalised phosphorylated to normalised total protein using ImageJ. Representative images of cropped blots (original blots are provided in the supplementary information). N ≥ 3 independent experiments. ANOVA with multiple comparisons test (Tukey’s), Mean ± SEM, * ≤ 0.05, ** ≤ 0.01, *** ≤ 0.001.

Data from the recent pandemic of SARS-CoV-2 (COVID-19) has revealed that pulmonary microangiopathy and microvascular dysfunction are responsible for much of the observed morbidity and mortality associated with COVID-19^47,48^. Of relevance to this study, it is now well established that a dysregulated cytokine immune response correlates with COVID-19 disease severity^49^. A key player in this cytokine response is IL-6, circulating levels of which are significantly elevated in COVID-19 patients, and as such its presence has been reported to contribute to the vascular pathology^50–52^. Although blocking IL-6 signalling as a therapeutic intervention has been extensively studied in other diseases^53,54^, randomised controlled clinical trials with biologics targeting the IL-6 receptor, including Tocilizumab, have already shown some evidence of clinical benefit in COVID-19 cases^55–58^. Notably, COVID-19 patients have also been found to exhibit significantly enhanced circulating levels of LRG1 that, along with other biomarkers, can distinguish mild from severe disease^38–41^. These data raise the possibility that IL-6 induces systemic and local upregulation of LRG1 in COVID-19 patients that will then exert its angiopathic role on the pulmonary microvasculature. We therefore sought to investigate whether blocking IL-6 signalling with Tocilizumab, a monoclonal antibody against the IL-6 receptor, affects *LRG1* gene expression in human pulmonary microvascular endothelial cells (HPMEC), where IL-6 treatment drives the gene and protein expression of LRG1 (Fig. 5a, Supplementary Fig. 2). Following IL-6 receptor blockade, we indeed observed a significant reduction in *LRG1* gene expression after 48 h, which was also reflected in the phosphorylation of STAT3 (Fig. 5a, b). In addition, we explored TGFβ signalling downstream of LRG1 in the lung endothelial cells, and interestingly, we observed that levels of phosphorylated SMAD1/5 followed a similar pattern to *LRG1* expression upon IL-6 stimulation and blockade (Fig. 5c), whereas no difference was seen at the levels of phosphorylated SMAD2 (Fig. 5d). This finding suggests that in addition to affecting pericytes, LRG1 might also promote its vascular corrupting effects through direct modification on the endothelial-specific TGFβ angiogenic switch driving it towards the proangiogenic phosphorylated SMAD1/5 pathway, similar to our previously published data^15^. These results suggest a potential pathogenic role for LRG1 in COVID-19 mediated damage to the lung and suggest that LRG1 can serve as both a prognostic marker for the severity of the disease, as well as a future therapeutic target.

## Discussion

LRG1 was initially identified as a new and potent angiogenic molecule that exerts its functions through modifying the TGFβ pathway^15^. Since then, the angiogenic and mitogenic roles of LRG1 have been well established in a range of different pathologies^14,15,17,18,44^. Although most studies involving LRG1 are focused on its downstream effectors, where the TGFβ pathway is frequently highlighted as a key player, only a few have explored the upstream signalling routes leading to LRG1 induction. Early evidence has suggested that the proinflammatory cytokines TNFα, IL-1β, and IL-6 can upregulate LRG1 expression either alone or synergistically^4,29–32^. In addition, LRG1 has been identified as a useful biomarker to distinguish between patients with active or inactive systemic juvenile idiopathic arthritis and in patients with rheumatoid arthritis during Tocilizumab treatment ^8,59^. Taking these reports into account, together with other studies where the IL-6/STAT3 axis has been implicated in the regulation of LRG1^20,60–62^ and a study showing that IL-6 promotes defective angiogenesis^34^, we explored the potential transcriptional regulation of *LRG1* by IL-6. We showed that IL-6 activated *LRG1* transcription to promote angiogenesis with reduced mural cell coverage through the phosphorylation and binding of STAT3 to its conserved consensus site on the *LRG1* gene promoter. In pathology, LRG1 is widely expressed by endothelial cells, but the IL-6 receptor is expressed at low levels in this cell type^63,64^, indicating that either low levels of IL-6 are sufficient to induce *LRG1* transcription or that trans-signalling mechanisms might be in place.

**Figure 6:**
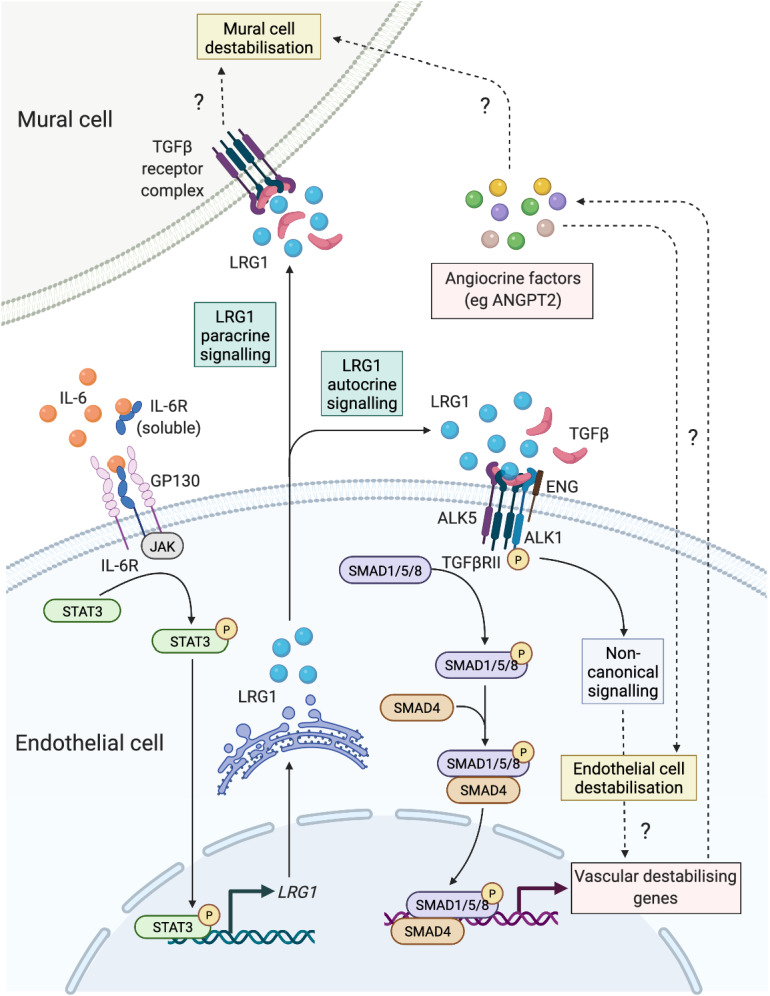
IL-6-dependent induction of LRG1 and proposed downstream angiopathic effector mechanisms. IL-6 induces LRG1 in endothelial cells, but not mural cells. LRG1 may then act in an autocrine loop on endothelial cells through the TGFβ receptor complex to activate canonical and non-canonical signalling that will modify endothelial cell function and induce vascular destabilising genes. In turn, the LRG1-mediated induction of angiocrine factors may result in indirect angiopathic effects on endothelial cells and mural cells. Alternatively, LRG1 may signal in a paracrine fashion directly on mural cells to drive destabilisation. Dashed lines with question marks represent speculative pathways. Created with Biorender.com.

In investigating the role of IL-6 in driving vascular dysfunction, Gopinathan et al.showed that IL-6 promotes angiogenesis with reduced pericyte coverage through the activation of Angpt2 and Jagged1 independently of VEGF signalling which, as the authors claim, leads to angiogenesis with good pericyte coverage through the phosphorylation of Erk and Dll4^34^. These findings are specific to endothelial cells and exhibit a quite different pattern to angiogenic signals that occur in cancer, where IL-6 and VEGF are interdependent due to the more complex signalling networks^65,66^. We have shown in the past that genetic ablation of *Lrg1* caused reduction in *Vegfa* expression in the mouse retina^15^, and others have demonstrated that LRG1 can induce VEGFA in colorectal cancer^67^. However, these two pathways have also been shown to be independent in the context of vascular dysfunction. Accordingly, a recent study highlighted that whilst VEGF and LRG1 are both angiogenic in diabetic nephropathy, they retain distinct roles in driving abnormal angiogenesis^68^, mostly due to the different downstream mediator pathways. In our RNAseq studies in mouse solid melanoma tumours, we have shown that genetic ablation of *Lrg1* did not affect the expression of *Vegfa* or any of its effector molecules^19^, providing further evidence that LRG1 and VEGF control pathological angiogenesis through independent mechanisms. These findings support the hypothesis that local induction of LRG1 is partly responsible for corrupting the physiological angiogenic process via local autocrine and paracrine signalling mechanisms (Fig. 6).

How IL-6-drives defective angiogenesis remains unclear but there is evidence that it may be mediated partly through modifying Notch and angiopoietin signalling^34^. Although Notch signalling has been extensively studied in the regulation of embryonic and tumour vascular development, the role of the Notch transcription factors *Hey1/Hes1* is not yet fully understood. Our data showed that only *Hey1* gene expression was altered in the IL-6-treated wild type and *Lrg1*^−/−^ metatarsals, and therefore more work is required to unravel the roles of Notch signalling in LRG1-dependent destabilised angiogenesis. The angiopoietin genes were also tested, with *Angpt2* found to be upregulated by IL-6 in wild type metatarsals, but not in those derived from *Lrg1*^−/−^ mice. ANGPT2 has been implicated in the detachment of pericytes from blood vessels causing dysfunctional and less mature vessels^69,70^ and opens up the possibility that *ANGPT2* may be regulated downstream of LRG1. This finding is in accordance with the reduction seen in the αSMA^+^ mural cell coverage in our data (Fig. 3c), confirming a possible causative role for Angpt2 in LRG1-mediated neovascularisation and vascular destabilisation (Fig. 6).

Pericytes and vascular smooth muscle cells, both defined as mural cells, exert similar roles in providing and regulating vascular integrity, with differences being partly determined by their positioning along the vascular tree. Although examining mural cell coverage has proven to be problematic due to the lack of specificity of expression markers, it is particularly important as it is directly related to vascular stability and function. TGFβ signalling retains a central role in regulating the differentiation of PDGFRβ^+^ progenitor cells to αSMA^+^, NG2^+^ and desmin^+^ cells, and αSMA^+^ perivascular cells are subsequently more sensitive to changes in TGFβ signalling than NG2^+^ and desmin^+^ cells^71^. In addition, altered endothelial TGFβ signalling has been shown to affect mural cell attachment and vessel stabilisation^72^. Consistent with these, our data shows that the αSMA^+^ cell population is predominantly affected by the endothelial IL-6-driven induction of LRG1 expression, either by responding indirectly to the altered LRG1-mediated endothelial TGFβ signalling or through endothelial cell secreted LRG1 acting directly on mural cell TGFβ signalling. Although the paracrine signalling seems to be the most plausible mechanism, alternative mechanisms where LRG1 impacts the proliferation and migration of mural cells or induces downstream expression of other angiocrine factors (eg. ANGPT2) cannot be excluded and require further investigation (Fig. 6).

The data described here are consistent with our previous study in cancer models where *Lrg1* deletion resulted in tumour vessel normalisation through increased mural cell coverage (αSMA^+^, NG2^+^) and improved basement membrane deposition^19^. However, as with our current study, variability was also seen among the αSMA^+^ and NG2^+^ mural cell populations, as well as in the different tumour models studied. IL-6 expression is high in the tumour microenvironment^73^, with a major source being from cancer-associated fibroblasts. It is likely, therefore, that the high LRG1 expression levels observed in tumour endothelium are also driven by IL-6 and result in the subsequent disruption of endothelial-mural cell associations and consequential abnormal vascularisation.

Aside from cancer, where vascular abnormality is a contributing factor to progression, vessel dysfunction also plays a central role in the pathophysiology and clinical manifestation of other diseases^13^. In COVID-19-associated disease, endothelial dysfunction, hypoxia, microvascular inflammation, and thrombosis, are thought to be triggered by significantly elevated levels of inflammatory cytokines, including IL-6^47,48,74,75^. Following recent findings, showing high circulating LRG1 levels in COVID-19 patients^38–40^, we propose that IL-6 is driving LRG1 production in the lung resulting in some of the pulmonary vasculopathy observed. We show here that blocking IL-6 signalling in pulmonary microvascular endothelial cells with Tocilizumab led to reduced *LRG1* levels thus attenuating the production of a significant angiopathic factor. Studies and meta-analyses on the use of Tocilizumab in the clinics to treat severe and critically ill COVID-19 patients have reported beneficial outcomes, although critics had initially questioned the efficiency of identifying the right COVID-19 positive populations to treat with Tocilizumab^76,77^. If LRG1 is angiopathic in COVID-19 patients, then inhibition of IL-6 may not be sufficient to stop LRG1 as IL-1β and TNFα, both prevalent in COVID-19 infected lungs^50^, are also capable of LRG1 induction^4^. Therefore, by targeting LRG1 directly, one may specifically inhibit the downstream pathogenic roles of LRG1 without impeding the homeostatic functions of IL-6.

To conclude, we have shown that LRG1 is a major driver of pathological angiogenesis, and that it disrupts normal vascular physiology through reducing mural cell coverage of newly developed vessels. It is highly likely that such effects aren’t restricted to new vessels but may also occur in existing vessels, causing destabilisation, increased permeability and priming the vessels for sprouting angiogenesis. These LRG1-driven effects may be transcriptionally regulated by IL-6, which induces the phosphorylation of STAT3 and its binding to the *LRG1* promoter to activate transcription (Fig. 6). Whilst IL-6 induced LRG1 may act in an autocrine fashion on endothelial cells, and therefore affect their function directly, our data demonstrates that mural cells also respond to LRG1. However, it is unclear if endothelial cell derived LRG1 acts in a paracrine manner on mural cells or via LRG1 driven expression of other angiocrine factors (Fig. 6). By directly blocking the downstream angiopathic molecule LRG1, either by using a function-blocking antibody or potentially by genetic manipulation, it may be possible to normalise the vascular phenotype. Our data highlight the potential of using LRG1 as a new therapeutic target to treat conditions with prominent pathological angiogenesis and dysfunctional vessels, such as age-related macular degeneration, cancer, and COVID-19-related pulmonary complications^44,78^.

## Methods

All experiments were approved by the Ethical Review Body of the UCL Institute of Ophthalmology. All experiments and procedures in mice were performed in compliance with the UK Animals Act and the Animal Welfare, as well as the ARRIVE guidelines.

### Cell culture

HUVEC were cultured in EGM2 media (Lonza, CC-3162) and were used between passages 2–6. Cells were serum-starved for 16 h in EBM-2 (Lonza, 00190860) supplemented with 0.1% FBS before being treated with 50 ng/ml IL-6 (R&D Systems, 206-IL-010/CF) for the indicated times.

Human pulmonary microvascular endothelial cells (HPMEC) (Promocell) were cultured in specific media (Promocell, C-22020) following the manufacturer’s instructions. Cells were serum starved for 16 h in media supplemented with 0.1% FBS before being treated, and were used between passages 2–6.

HepG2 cells were grown in EMEM media (ATCC, 30-2003) supplemented with 10% FBS. The cells were serum-starved in 0.1% FBS before being treated with 50 ng/ml IL-6 (R&D Systems, 206-IL-010/CF) for the indicated times.

Mouse brain microvascular endothelial cells (MBMEC) were isolated from adult wild type C57Bl6 mouse brain tissue, as previously published^79^. Briefly, the brain tissue was homogenised in HBSS media, supplemented with 10 mM HEPES, 0.1% P/S, 0.5% BSA, and centrifuged. The pellet was mixed with 25 ml ice-cold 22% BSA, centrifuged, and resuspended again before passing through a 70 μm pore cell strainer. Brain microvessels trapped on the strainer were then subjected to collagenase type I and dispase enzyme digestion for 60–90 min with frequent shaking at 37 °C. The solution was then centrifuged, and the endothelial cells were resuspended in EGM-2 supplemented with 0.1% puromycin before plating on fibronectin-coated plates. The media was changed every two days, and the cells were used when they reached confluence. Brain microvascular pericytes were isolated using the same protocol but were cultured in EGM-2 without puromycin. The absence of endothelial cells in the pericyte cultures was confirmed by staining for CD31 and αSMA markers.

### Metatarsal angiogenesis assay

The metatarsal angiogenesis assay was performed as previously described^80^. Metatarsal bones were dissected from wild type C57Bl6 or *Lrg1*^−/−^ embryos at E17.5 and cultured in 0.5% porcine gelatin-coated plates for a total of 11 days in MEMα (Thermo Fisher Scientific, 31985070) supplemented with 10% FBS and 1% P/S, and various treatments including 50 ng/ml IL-6 (R&D Systems, 206-IL-010/CF), and 70 μg/ml recombinant human LRG1 protein produced in-house^44^. On day 11, metatarsals were fixed in 4% PFA, blocked and permeabilised in 10% BSA with 0.1% Triton for 1 h at room temperature, and stained overnight at 4 °C with anti-CD31 (1:100, BD Pharmingen, 553370), anti-αSMA (1:100, Sigma, C6198) or anti-NG2 (1:100, Millipore, AB5320) antibodies. The fluorophore labelled secondary antibodies were incubated for 2 h at room temperature, and the bones were then stored in PBS until imaging. For analysis of angiogenesis, images were taken using a Nikon epifluorescence microscope with 4 × objective as tile scans to include the whole CD31^+^ vessel area. Total tubule length and number of junctions were measured using Angiosys software (TCS Cellworks) after manual thresholding. For confocal imaging, metatarsals were cultured on coverslips, then mounted on glass slides, and imaged using maximum intensity projections (ZEISS LSM 700 confocal microscope). 3D renders were generated using IMARIS software from imported Z stacks.

### Aortic ring angiogenesis assay

Aortic vessels were dissected from young adult (2–4 months old) wild type C57Bl6 or *Lrg1*^−/−^ animals and kept in cold Optimem while the fat tissue and the branching vessels were carefully removed under a dissection microscope. The blood inside the aortic tube was flushed out before it was sliced into rings of ~ 0.5-1 mm. The aortic rings were serum-starved in Optimem + 1% P/S overnight at 37 °C, then plated in 50 µl of 1 mg/ml Rat Tail Collagen I (Thermo Fisher Scientific, A1048301) per well of a 96-well plate. The aortic rings were cultured for seven days in Optimem supplemented with 10% FBS and 1%P/S, and various treatments including 50 ng/ml IL-6 (R&D Systems, 206-IL-010/CF) and 70 μg/ml recombinant human LRG1 protein produced in-house. Media and treatments were changed every 2–3 days. On day 7, the rings were washed with PBS and fixed in 4% PFA for 10 min at room temperature. PFA was washed off, and the cultures were blocked and permeabilised in 5% goat serum with 0.5% TritonX for 1 h at room temperature. Isolectin B4 (Vector Labs, FL-1201) was used to stain the endothelial sprouts, and anti-αSMA (Sigma, C6198) and anti-NG2 (Millipore, AB5320) antibodies for the pericytes. Primary antibodies and Isolectin B4 were incubated overnight at 4 °C. Fluorophore labelled secondary antibody incubation was performed for 2 h at room temperature, and the aortic rings were kept in PBS at 4 °C until imaging. For sprouting analysis, images of the whole aorta were taken using a Nikon Epifluorescence microscope with a 4 × objective as tile-scans and stacks. The numbers of sprouts were measured manually in ImageJ.

### Mural cell coverage (metatarsal bones, aortic rings)

Explant tissues were cultured, washed, and stained using standard protocols as mentioned above. For the pericyte coverage analysis, the endothelium in the metatarsal assay was stained with an anti-CD31 (BD Pharmingen, 553370) antibody and in the aortic ring assay with Isolectin B4 (Vector Labs, FL-1201). Pericytes were stained with anti-αSMA (Sigma, C6198) and anti-NG2 (Millipore, AB5320) antibodies. Single images were taken using a 20 × objective focussed on the plane of vessels using a Nikon epifluorescent microscope. For quantitative analysis, using NIS Elements Software, single thresholds were calculated for each individual channel (endothelium, pericytes) and the intersection of the endothelium covered by pericytes. The analysis was blinded to treatments and was performed on masked images with different thresholds used per image as required to avoid saturation. Pericyte coverage was then calculated as % (Intersection/Endothelium), and the values were normalised to the average of the internal control (vehicle/PBS) for every experiment. The normalised values of at least three independent experiments were pooled for statistical analysis. Outliers were eliminated using the average ± 2SD mean.

### RT-qPCR

Cells were washed in PBS and lysed in RLT buffer (Qiagen) to isolate total RNA. 1 μg of total RNA was reverse-transcribed to generate cDNA (NEB, E3010), and then used for RT-qPCR (NEB, M3003) to amplify the mouse and human *LRG1* genes (mouse *Lrg1*: forward primer: CCATGTCAGTGTGCAGATTC, reverse primer: AAGAGTGAGAGGTGGAAGAG, human *LRG1*: forward primer: CAGCGACCAAAAAGCCCAG, reverse primer: ATTTCGGCAGGTGGTTGACA). Relative gene expression was normalised to the housekeeping mouse *Gapdh* gene (forward primer: ACTGAGGACCAGGTTGTCTCC, reverse primer: CTGTAGCCGTATTCATTGTCATACC) and the human *HPRT* gene (forward primer: TGACACTGGCAAAACAATG, reverse primer: GGTCCTTTTCACCAGCAAG).

### Cloning-luciferase assays

A 982 bp genomic region spanning the human *LRG1* gene promoter (chr19: 4,540,011-4,540,992, UCSC, Human GRCh37/hg19 Assembly) was amplified from human genomic DNA and cloned into the pGL4.10 luciferase vector (hLRG1prom-full) by nested PCR. A 213 bp region directly upstream of the transcription start site that spanned the STAT3 consensus binding site was removed by PCR (hLRG1prom-F6). To further explore the impact of STAT3 binding on LRG1 transcription, a third version of the vector was created that lacked 54 bp spanning the STAT3 consensus binding region (hLRG1prom-STAT3). 30,000 HepG2 cells per well were plated in a 96-well plate and were serum-starved for 24 h before transfection. 160 ng of each luciferase vector was transfected together with 40 ng of a Renilla luciferase vector, which served as an internal control using Lipofectamine 2000. The cells were treated with 50 ng/ml IL-6 for 24 h after transfection, and a further 24 h were allowed before the cells were lysed, and luciferase assays were performed (Promega, Dual-Luciferase Reporter Assay System E1910). Luciferase activity was measured as relative luciferase units (RLU) and was normalised to the internal control (Renilla) and then to the vehicle within each experiment.

### Chromatin immunoprecipitation assay

Chromatin Immunoprecipitation assays were performed following the manufacturer’s protocol (Sigma, 17-10086). HepG2 cells were stimulated with 50 ng/ml IL-6 in complete media (10%FBS-EMEM) for 16 h before proteins were cross-linked with 1% (final) formaldehyde for 10 min at room temperature. Cell lysates were sonicated using a standard probe sonicator for 4 × 15 s with 45 s intervals at 10 microns amplitude to generate chromatin fragments with an average size of 100–600 bp. Immunoprecipitation binding reactions were performed using the following antibodies: anti-RNA polymerase II (Cell Signalling, 13546) and anti-phosphorylated STAT3 (Cell Signalling, 9145). 1 μg of normal rabbit IgG (Cell Signalling, 2729) was used as a negative control and 5% of chromatin was used as input material. Immunoprecipitated DNA was amplified by RT-qPCR using primers specific for the human *LRG1* promoter (forward primer: AATCAGGAAGCAAGGAAAGA, reverse primer: CCGTCCAGTTCCTGATAGG) and the data were analysed using the ΔΔCt method. Data are presented as fold-enrichment to IgG.

### Tocilizumab

HPMEC were serum-starved overnight and then treated with vehicle (PBS), 50 ng/ml IL-6 alone or in combination with 20 μg/ml anti-IL-6R Tocilizumab (Toc) antibody (Novus Biotechne (NBP2-75192) for 24 or 48 h. RNA and protein were collected, and human *LRG1* gene expression was assessed by RT-qPCR. Total cell lysates were used to determine the expression of the phosphorylated and total STAT3, SMAD1/5, SMAD1, SMAD2, SMAD2/3 proteins (Cell Signalling), and densitometric analysis was performed using ImageJ.

### Protein preparation, acetone precipitation, western blotting

Cells were washed and lysed on ice with RIPA buffer supplemented with protease and phosphatase inhibitors. Lysates were cleared by centrifugation at maximum speed (21,000×*g*) for 10 min at 4 °C, before being subjected to SDS‐PAGE and Western blotting^81^. Cell culture supernatants were collected, diluted with ice-cold acetone at a ratio of 1:4 and were left at − 20 °C overnight^82^. The day after, the suspension was centrifuged at (21,000×*g*) for 10 min at 4^o^C^82^. The pellet was left to air-dry for 30 min at room temperature before being resuspended in water and sample buffer and subjected to SDS-PAGE and Western blotting^81^. Secreted LRG1 protein levels were normalised to total cell protein stained by Ponceau S. Specific antibodies (Cell Signalling) against P-STAT3 (cs#9145), total-STAT3 (cs#9139), P-SMAD2 (cs#3108), total-SMAD2/3 (cs# 8685), P-SMAD1/5 (cs#9516), total-SMAD1 (cs#6944), and LRG1 (ProteinTech, 13224-1-AP) were used. GAPDH served as a housekeeping gene. Densitometry analysis was performed using ImageJ^83^.

## Supplementary Information

Supplementary Information.

## Data Availability

All data generated or analysed during this study are included in this published article (and its Supplementary Information file).
