# Publisher Correction: Angiopathic activity of LRG1 is induced by the IL-6/STAT3 pathway

**DOI:** 10.1038/s41598-022-09460-x

**Published:** 2022-03-29

**Authors:** Athina Dritsoula, Laura Dowsett, Camilla Pilotti, Marie N. O’Connor, Stephen E. Moss, John Greenwood

**Affiliations:** Institute of Ophthalmology, University College London, 11-43 Bath Street, London, EC1V 9EL UK

Correction to: *Scientific Reports* 10.1038/s41598-022-08516-2, published online 22 March 2022

The original version of this Article contained an error in Figure 6 where the red circles around the question marks were incorrectly added in the published figure.

**Figure 6:**
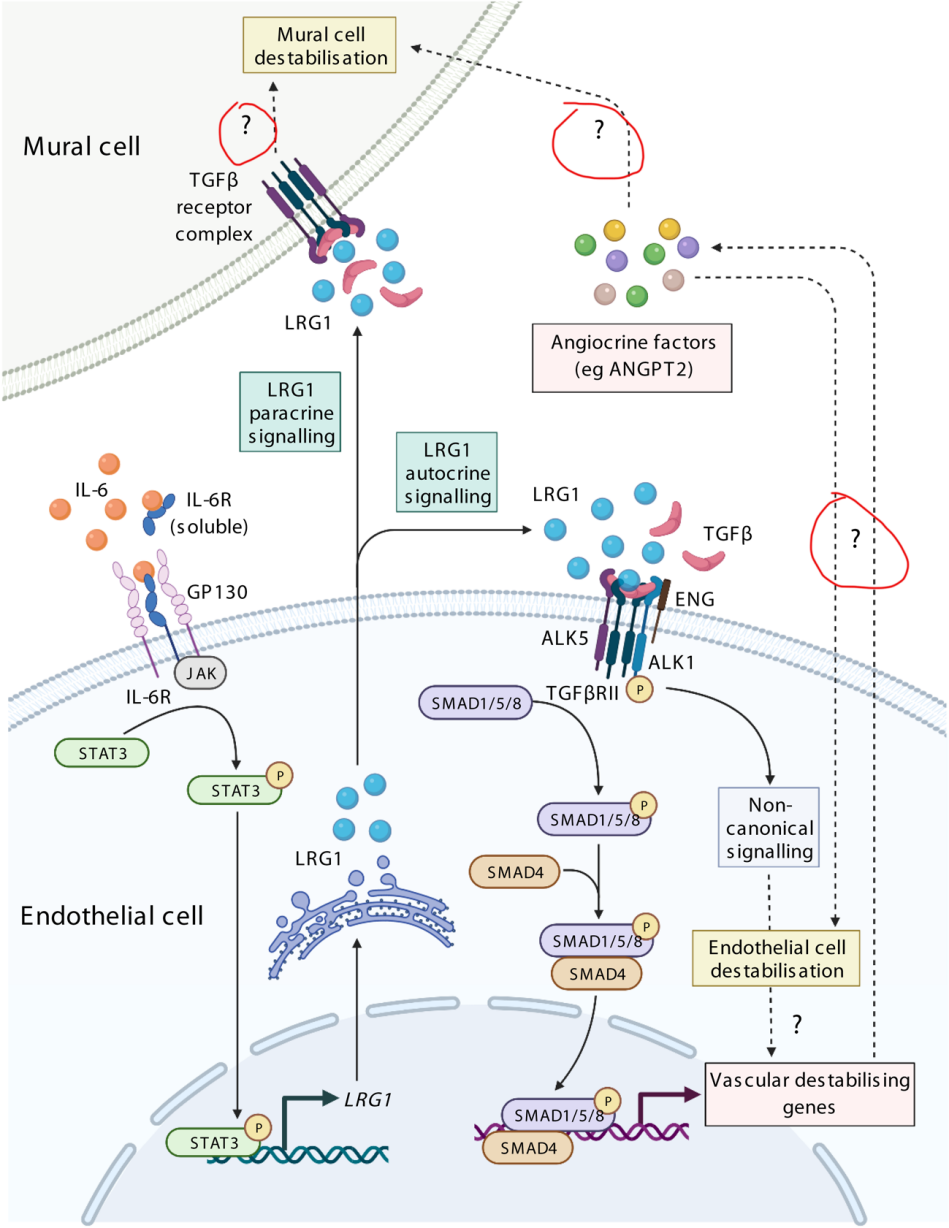
IL-6-dependent induction of LRG1 and proposed downstream angiopathic effector mechanisms. IL-6 induces LRG1 in endothelial cells, but not mural cells. LRG1 may then act in an autocrine loop on endothelial cells through the TGFβ receptor complex to activate canonical and non-canonical signalling that will modify endothelial cell function and induce vascular destabilising genes. In turn, the LRG1-mediated induction of angiocrine factors may result in indirect angiopathic effects on endothelial cells and mural cells. Alternatively, LRG1 may signal in a paracrine fashion directly on mural cells to drive destabilisation. Dashed lines with question marks represent speculative pathways. Created with Biorender.com.

The original Figure 6 and accompanying legend appear below.

The original Article has been corrected.

