# Supplementary material for: Angiopathic activity of LRG1 is induced by the IL-6/STAT3 pathway
Source: Sci Rep. 2022 Mar 22;12:4867. doi: 10.1038/s41598-022-08516-2 (PMC8938720; doi:10.1038/s41598-022-08516-2)

Supplementary Information

Angiopathic activity of LRG1 is induced by the IL-6/STAT3 pathway

Athina Dritsoula[1]*, Laura Dowsett[1], Camilla Pilotti[1], Marie N. O'Connor[1], Stephen E Moss[1, 2], John Greenwood[1, 2]

[1] Institute of Ophthalmology, University College London, London, UK, [2] Equal senior authors

***Corresponding author: Athina Dritsoula, UCL Institute of Ophthalmology, 11-43 Bath Street, EC1V 9EL, London, UK**

Supplementary Figure 1

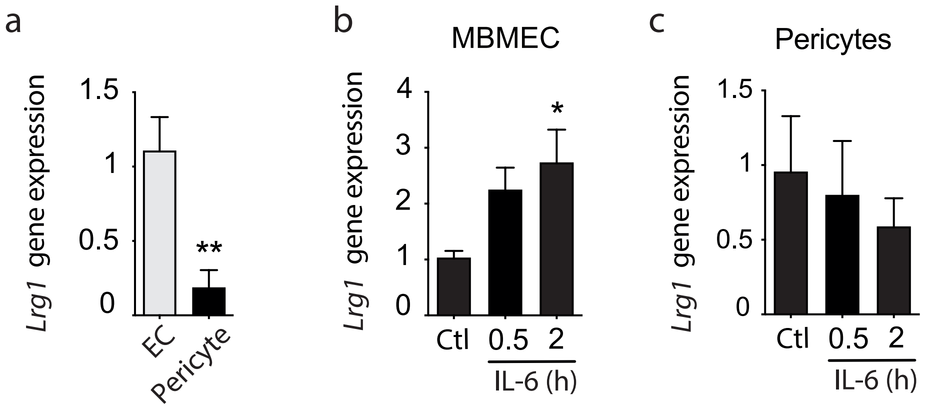

Supplementary Figure 1. a. The *Lrg1* gene is expressed in mouse brain microvascular endothelial cells (EC/MBMEC) but not in pericytes (from the same source). IL-6 induces expression of *Lrg1* in MBMEC (b) but has no effect in pericytes (c). Unpaired t-test (a), ANOVA (b). N≥3 independent experiments. Mean ± SEM, *≤0.05, **≤0.01

Supplementary Figure 2

a

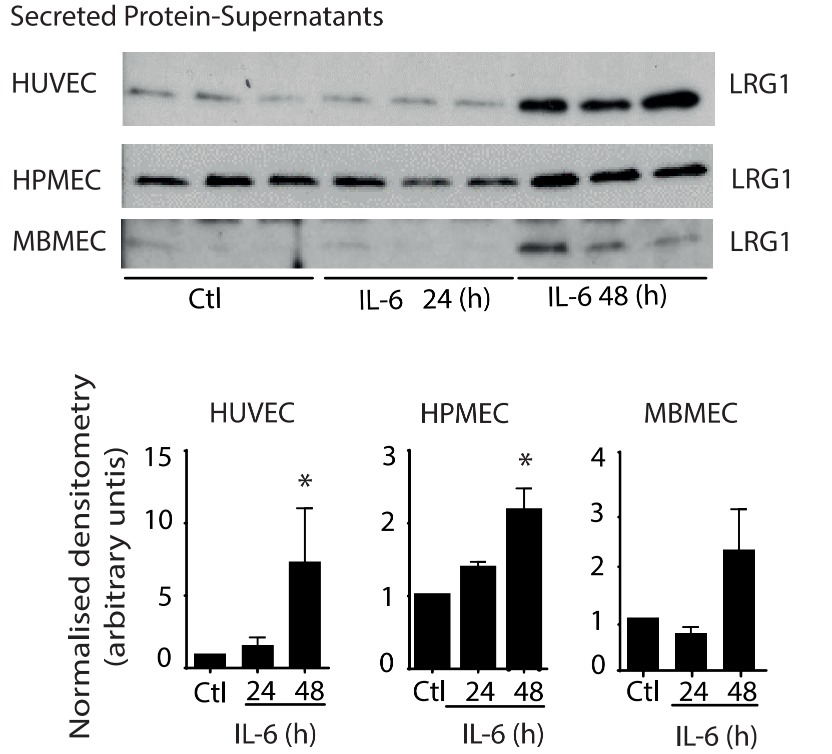

b

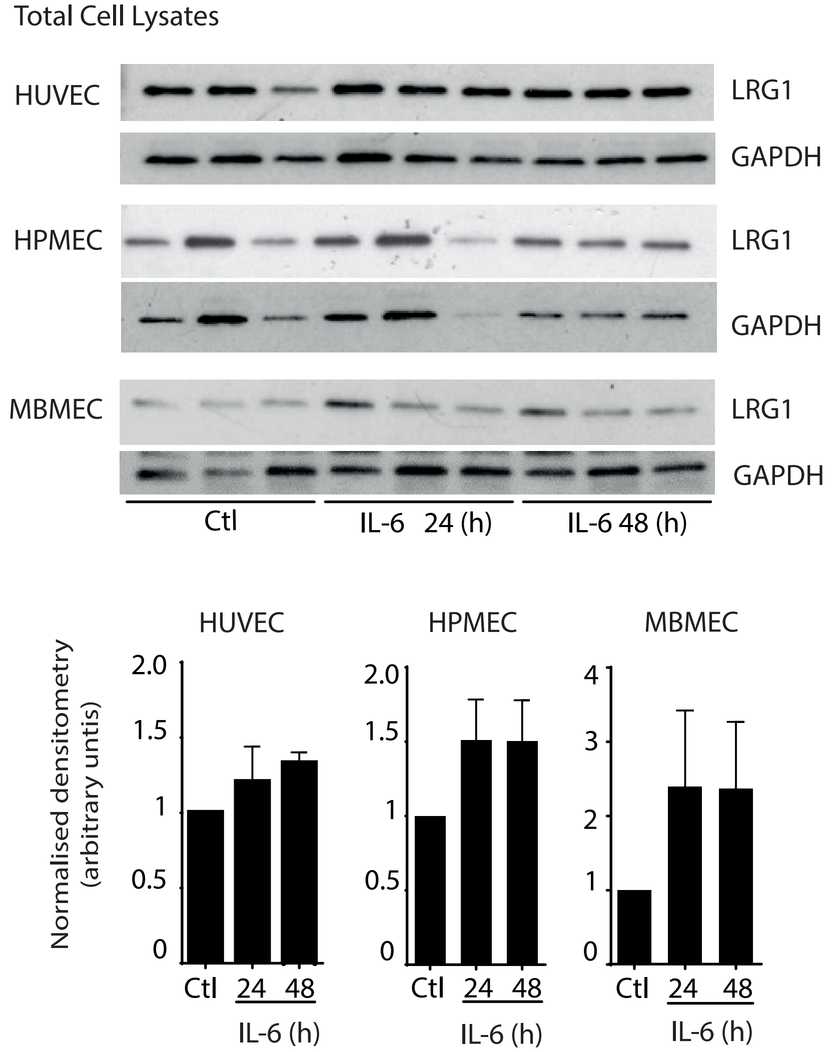

Supplementary Figure 2. IL-6 stimulation increases LRG1 protein expression. HUVEC, MBMEC and HPMEC were treated with PBS as control (Ctl) or with 50ng/ml IL-6 for 24 or 48h. a. Cell culture supernatants were collected and subjected to acetone precipitation and western blotting. Amounts of secreted LRG1 were normalised to total protein by Ponceau S staining. Histograms of normalised densitometry for secreted LRG1 in MBMEC and HUVEC are repeated here after main Figure 2 for clarity. b. Total cell lysates were prepared and LRG1 protein was assessed by western blotting and normalised to GAPDH. Densitometric analysis was performed using ImageJ. Kruskal-Wallis with Dunn's multiple comparisons test. N≥3 independent experiments. Mean ± SEM, *≤0.05

Supplementary Figure 3

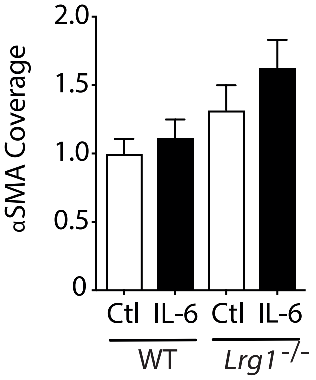

Supplementary Figure 3. αSMA$^+$ mural cell coverage in the aortic ring assay. Image analysis of aortic rings obtained from wild type or *Lrg1*$^{-/-}$ animals treated with PBS as control (Ctl) or 50ng/ml IL-6 and stained for Isolectin B4 and αSMA. Kruskal-Wallis with Dunn's multiple comparisons test. N≥3 independent experiments. Mean ± SEM.

Supplementary Figure 4

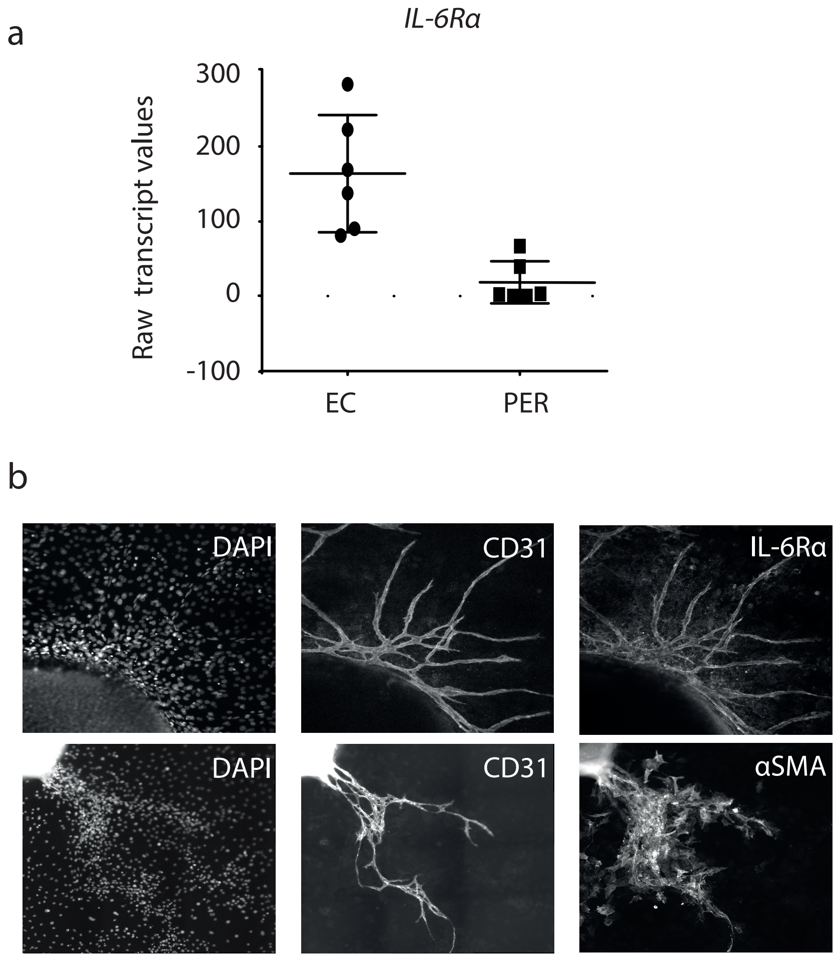

Supplementary Figure 4. a. Expression profiling by high throughput sequencing showing levels of expression for the *IL-6Rα* gene (raw copy number of transcripts) in endothelial cells (EC) and pericytes (PER) isolated from healthy mouse lung tissue. Data source: GSE162354. b. Metatarsal cultures stained for the endothelial marker CD31, the mural cell marker αSMA and for IL-6Rα. IL-6Rα staining co-colocalises with endothelial staining but exhibits a different pattern to the αSMA mural staining, consistent with endothelial, but not mural cell, expression. Mean ± SEM. Images were taken by a NIKON epifluorescence microscope at 20x objective.

Supplementary Figure 5

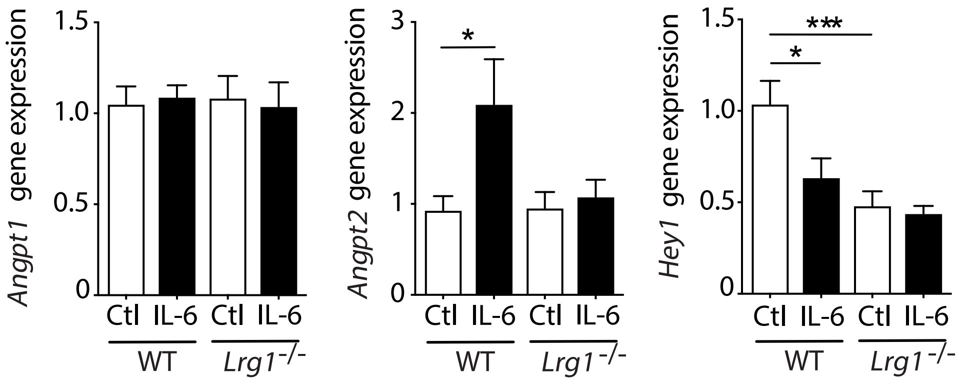

Supplementary Figure 5. Relative expression of *Angpt1, Angpt2,* and *Hey1* normalised to *Rpl13* housekeeping gene. RNA was isolated from metatarsal cultures obtained from wild type and *Lrg1*$^{-/-}$ mice and treated with 50ng/ml IL-6 or PBS as control (Ctl). N≥3. 1-way ANOVA with correction for multiple comparisons. Mean ± SEM. *≤0.05, ***≤0.001.

Supplementary Figure 6

a

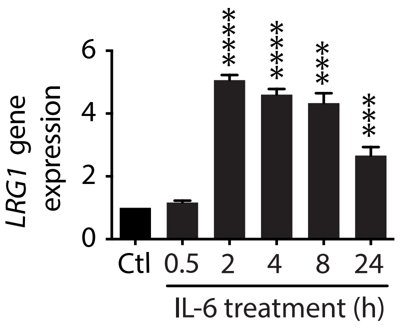

b

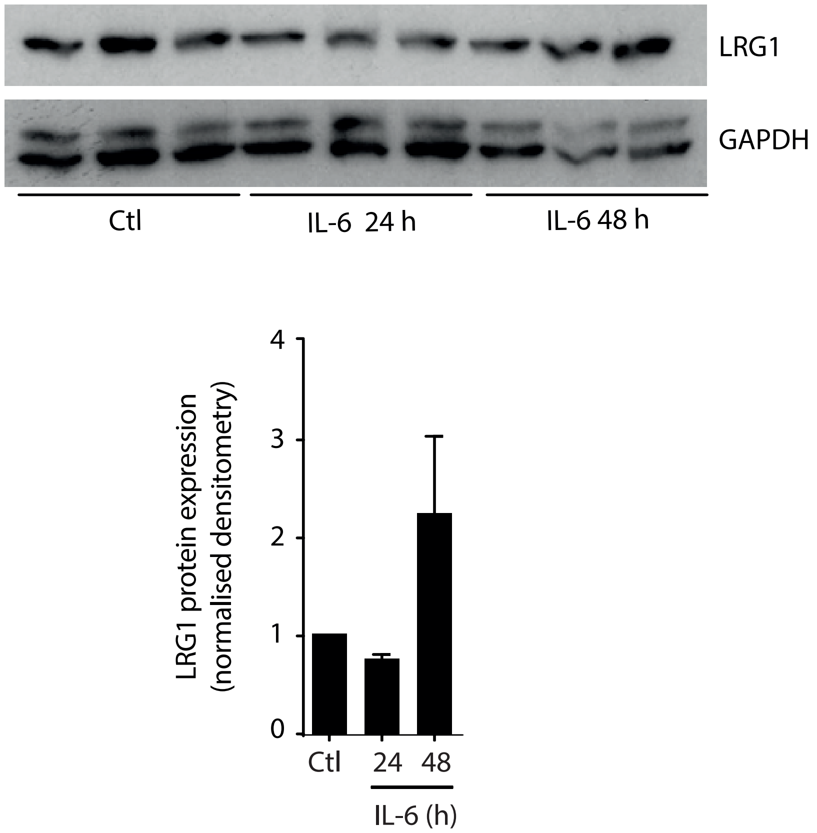

Supplementary Figure 6. IL-6 stimulation increases gene and protein expression of LRG1 in HepG2 cells. HepG2 cells were treated with PBS as control (Ctl) or 50ng/ml IL-6 for 24 or 48h. a. RNA was isolated and gene expression of *LRG1* was assessed by RT-qPCR normalised to *HPRT* housekeeping gene. ANOVA with Dunnett's multiple comparisons test. b. Total cell lysates were also prepared and LRG1 protein was assessed by western blotting and normalised to GAPDH. Densitometric analysis was performed using ImageJ. Kruskal-Wallis with Dunn's multiple comparisons test. N≥3 independent experiments. Mean ± SEM, ***≤0.001, ****≤0.0001

Supplementary Figure 7

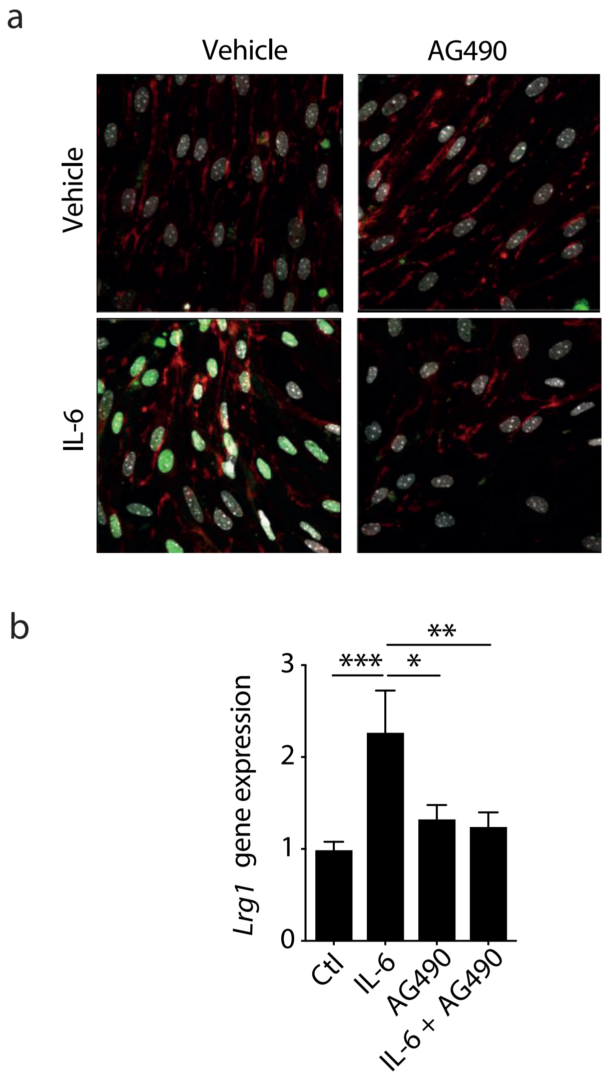

Supplementary Figure 7. JAK-2 inhibitor AG490 blocks *Lrg1* expression by pharmacologically inhibiting the JAK/STAT signalling pathway. Phosphorylation of STAT3 (a) and *Lrg1* gene expression (b) in MBMEC stimulated with IL-6 or vehicle and treated with AG490. MBMEC were serum-starved for 5 h before being treated with 50ng/ml IL-6 and 50μM AG490 for 20 min (a) or 2 h (b). For immunofluorescence staining cells were fixed in 4% PFA for 3 min followed by methanol permeabilisation for 3 min, before being stained with CD31 (Red) and P-STAT3 (Green). ANOVA with Tukey's multiple comparisons test. N≥3 independent experiments. Mean ± SEM, *≤0.05, **≤0.01, ***≤0.001

ORIGINAL BLOTS

Fig. 5b

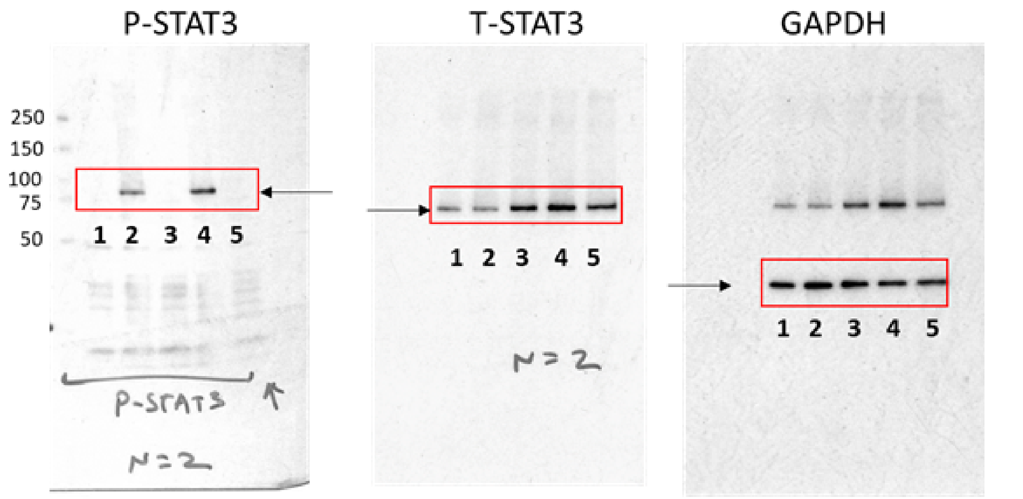

Fig. 5c

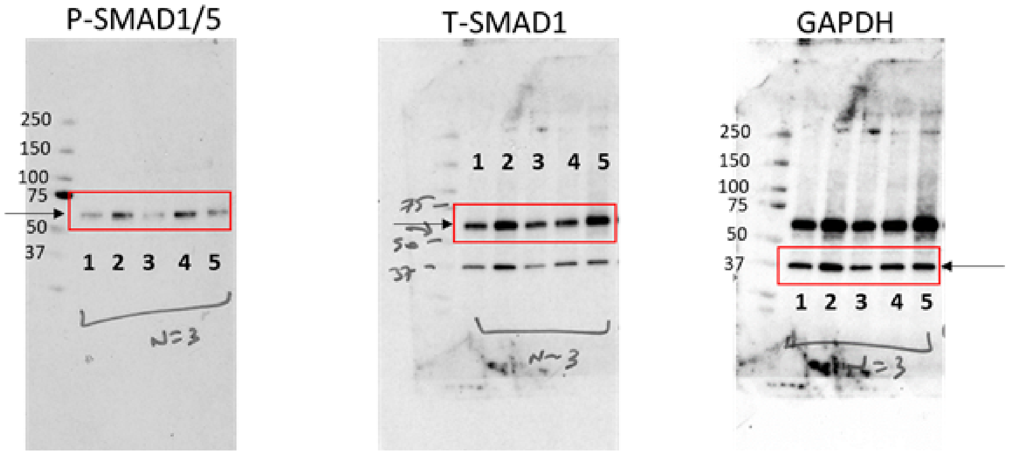

Fig. 5d

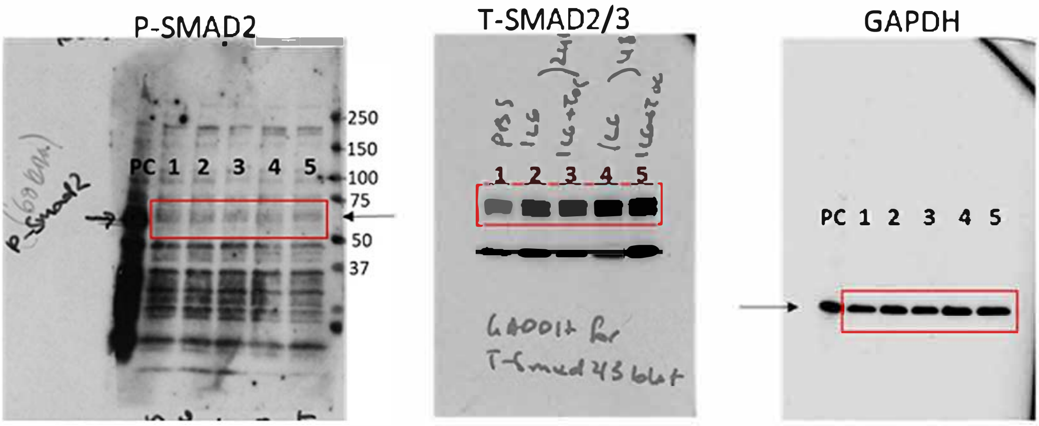

Supplementary Fig. 2a

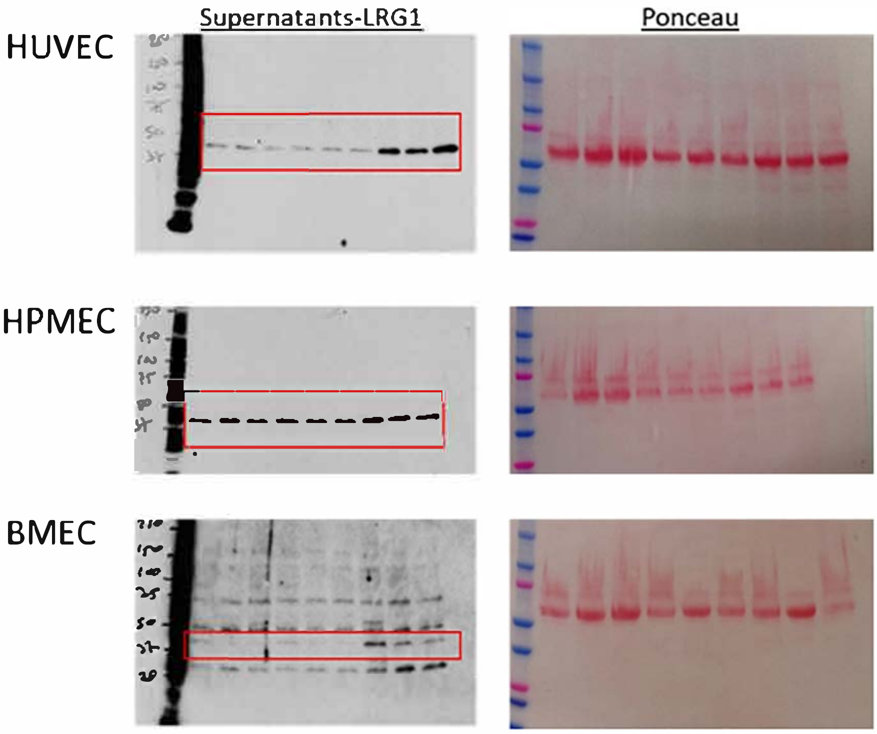

Supplementary Fig. 2b

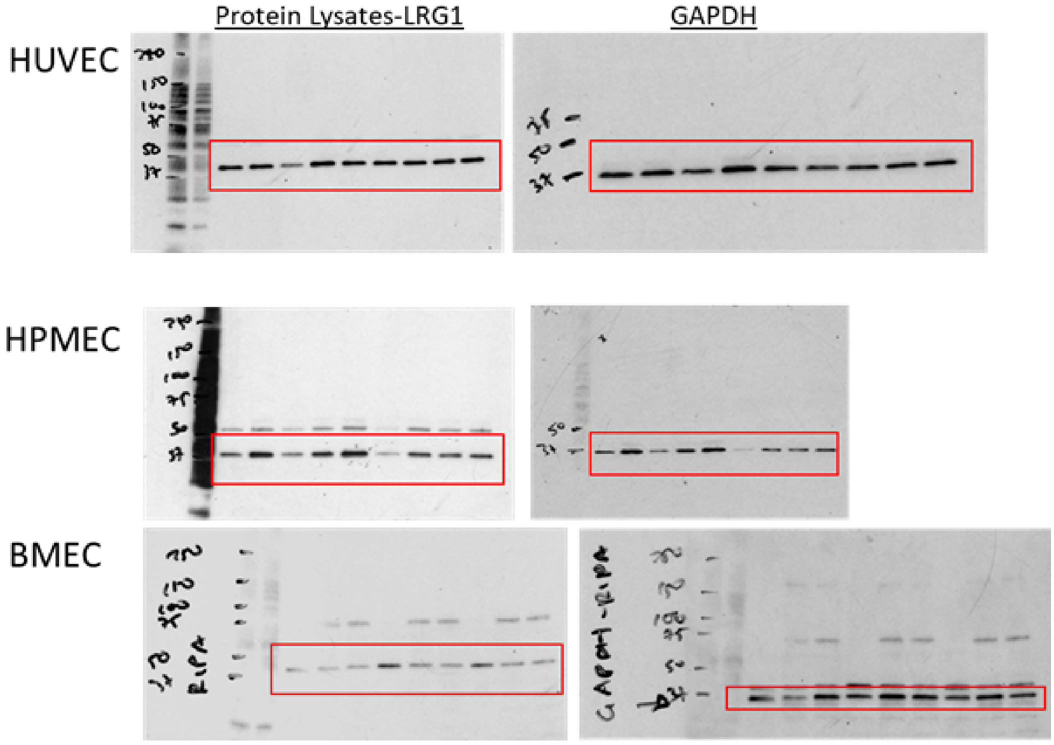

Supplementary Fig. 6b

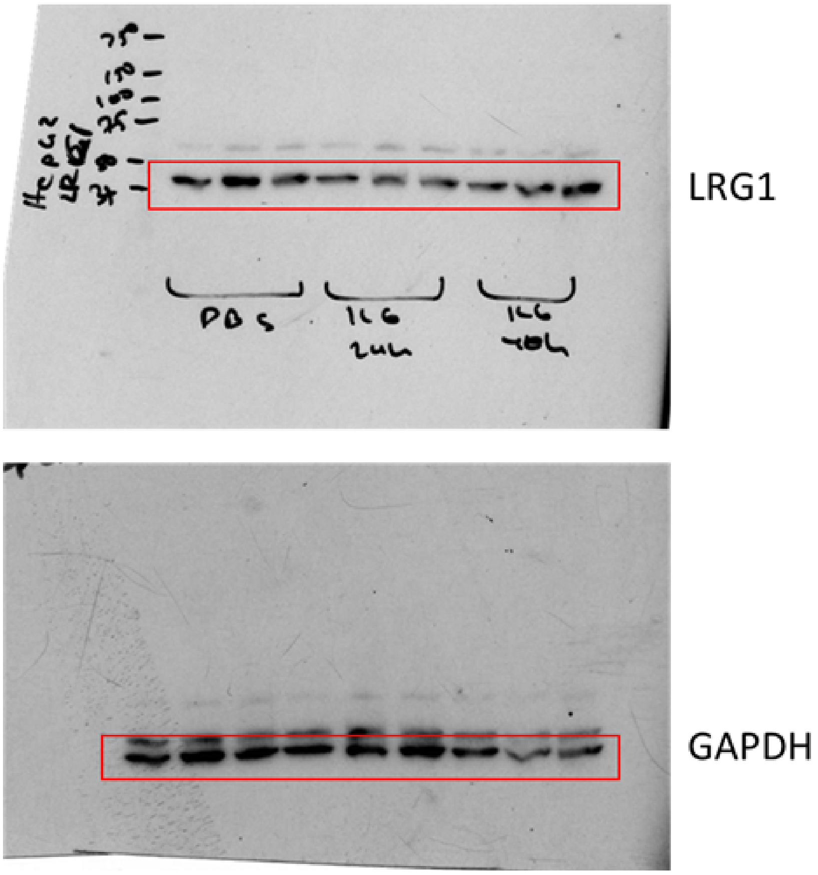

Supplement: Supplementary file 1 — Supplementary Information. [file 41598_2022_8516_MOESM1_ESM.pdf]
